# Implications of Discordance in World Health Organization 1997 and 2009 Dengue Classifications in Adult Dengue

**DOI:** 10.1371/journal.pone.0060946

**Published:** 2013-04-01

**Authors:** Victor C. Gan, David C. Lye, Tun L. Thein, Frederico Dimatatac, Adriana S. Tan, Yee-Sin Leo

**Affiliations:** 1 Communicable Disease Center, Tan Tock Seng Hospital, Singapore, Singapore; 2 Department of Medicine, National University of Singapore, Singapore, Singapore; University of Texas Medical Branch, United States of America

## Abstract

**Background:**

Revised dengue guidelines were published by the World Health Organization (WHO) in 2009 addressing severe dengue cases not classified by dengue hemorrhagic fever (DHF) and shock syndrome (DSS).

**Methods and Principal Findings:**

We conducted a retrospective cohort study to compare WHO 2009 and 1997 classifications using 1278 adult dengue cases confirmed by polymerase chain reaction assay from Singapore epidemics in 2004 and 2007 (predominantly serotype 1 and 2 respectively).DHF occurred in 14.3%, DSS 2.7% and severe dengue 16.0%. The two WHO dengue classifications were discordant in defining severe disease (p<0.001). Five DSS patients (15%) were classified as non-severe dengue without warning signs. Of severe dengue patients, 107 did not fulfil DHF criteria. Of these, 14.9% had self-resolving isolated elevated aminotransferases, 18.7% gastrointestinal bleeding without hemodynamic compromise and 56.1% plasma leakage with isolated tachycardia. We compared both guidelines against requirement for intensive care including the single death in this series: all six had severe dengue; only four had DHF as two lacked bleeding manifestations but had plasma leakage. Increasing length of hospitalization was noted among severe cases with both classifications but the trend was only statistically significant for WHO 2009. Length of hospitalization was significantly longer for severe plasma leakage compared with severe bleeding or organ impairment. Requirement for hospitalization increased using WHO 2009 from 17.0% to 51.3%.

**Conclusions:**

While the WHO 2009 dengue classification is clinically useful, we propose retaining criteria for plasma leakage and hemodynamic compromise from WHO 1997, and refining definitions of severe bleeding and organ impairment to improve clinical relevance having found that differences in these accounted for the discordance between classifications. Findings from our retrospective study may be limited by the study site - a tertiary referral center in a hyperendemic country - and should be evaluated in a wider range of geographic settings.

## Introduction

Dengue potentially affects more than 2.5 billion people primarily in the tropics and subtropics and is of increasing public health importance [1]. The World Health Organization (WHO) 1997 dengue guideline [2] based on a pediatric syndrome with a high mortality rate in Thailand and the Philippines from the 1950s emphasized the role of plasma leakage in the pathophysiology of dengue hemorrhagic fever (DHF) and dengue shock syndrome (DSS). The epidemiology of dengue in Singapore has shifted to older cases, possibly due to a prolonged period of vector control that led to increased dengue-susceptibles in an aging population [3]–[5]. Additionally, atypical presentations of dengue not attributable to plasma leakage have been reported, such as gastrointestinal complications [6] and encephalitis [7]. Some of these issues have been addressed by the WHO SEARO 2011 guidelines which was a regional update that included an additional category of ‘Expanded Dengue Syndrome’ among other modifications to the classification [8]. As this was a regional update, we did not use this in our study.

The WHO 2009 guideline [9] emphasized clinical triage [10] instead of a pathophysiologically defined syndrome. A broader range of symptoms was used to identify probable dengue cases. Severe dengue now encompasses one of three categories: shock from plasma leakage, clinically severe bleeding, or organ failure. In contrast, the WHO 1997 guideline defined DSS as the concomitant presence of bleeding, thrombocytopenia, plasma leakage and hemodynamic compromise. These changes carry public health and clinical care implications. It is crucial to examine the applicability of these two guidelines in different settings and their implications for clinical management of adult dengue. We hope in this study to address this problem by describing the overall epidemiological pattern of adult dengue cases in a single center when classified using both these systems. In particular we examine severe cases requiring intensive care or resulting in death, and examine impact on length of hospitalization. We investigate discordances between the classifications.

Previous evaluations of the WHO 2009 case definitions were performed using pediatric cohorts with mixed results: when compared with intensive care as a gold standard, an Indonesian cohort demonstrated WHO 2009 criteria to be significantly less sensitive but more specific [11], while a Nicaraguan cohort showed that WHO 2009 was significantly more sensitive with similar specificity [12]. A Thai study using a pediatric cohort did not analyze the performance of WHO criteria against intensive care [13]. The DENCO study used to derive WHO 2009 guidelines adopted various levels of nursing care and a mix of other interventions with the most severe category not yet amounting to intensive care as an objective criterion of clinical severity [14], which is open to criticism by Kalayanarooj [13] and Srikiatkhachorn et al [15]. We agree with Rigau-Perez that “To determine if the new definitions might have undesirable consequences on the quality of surveillance data, their sensitivity and specificity should be tested in a well-studied population of patients, or at least applied retrospectively to data from well-examined groups” especially from areas with different incidences of DHF [16]. We made use of the standardized clinical data available from the clinical care path used in dengue management in a tertiary infectious disease referral center in Singapore over two separate epidemic years to provide an analysis of the impact of using the different guidelines. Though prospective evaluation is optimal and we are currently pursuing such a study, well-collected retrospective data remains important in the interim to guide future research, especially when such analyses are not yet readily available.

This study aims to evaluate the latest WHO guideline using laboratory confirmed adult dengue cases in two epidemics in Singapore: 2004 (predominantly dengue serotype 1 [DENV-1]) and 2007 (predominantly dengue serotype 2 [DENV-2]) [17]. Comparison is made with the WHO 1997 guideline.

## Methods

### Ethics statement

The National Healthcare Group Domain Specific Review Boards granted ethics approval of the study with a waiver of informed consent for collection of anonymized case note data (DSRB B/05/115, DSRB E/08/567).

### Study population

We conducted a retrospective cohort study of all 1278 adult dengue cases confirmed by reverse-transcriptase polymerase chain reaction (RT-PCR) [18] in 2004 and 2007 and treated at the Communicable Disease Center (CDC), Singapore. All patients were managed using a standardized hospital dengue care path which improved clinical, treatment and outcome data consistency. Hospital electronic medical records were used for extraction of administrative, laboratory, microbiological and radiological data. Data extraction was performed by medically-trained research assistants. Rule-based data validation was performed for the entire data set. In addition, 10% of the cases were randomly selected for repeat data entry by another research assistant; data discrepancy was resolved by independent medical case note review by one of the authors. Patients were classified into the different WHO 1997 and 2009 dengue severity categories with available clinical, laboratory and radiological data from the entire clinical course till hospital discharge for inpatients and end of acute follow up for outpatients with strict application of the two WHO classifications.

### WHO 1997 classification [2]

Classification as dengue fever (DF) required the presence of fever and two or more of the following: headache, retro-orbital pain, myalgia, arthralgia, rash, leukopenia, or hemorrhagic manifestations. The tourniquet test was not performed. Diagnosis of DHF required fever and all three of: hemorrhagic tendencies; thrombocytopenia (platelet <100 000/mm^3^); and evidence of plasma leakage (hematocrit change of ≥20% or clinical fluid accumulation or hypoproteinemia [serum protein <63 g/dL]). For DSS, DHF cases required either (i) tachycardia (pulse >100/minute) with narrow pulse pressure (<20 mmHg) or (ii) hypotension for age (systolic blood pressure <90 mmHg).

### WHO 2009 classification [9]

Classification as probable dengue required fever with two or more of: nausea/vomiting, rash, aches/pains, leukopenia and any warning sign. Warning signs used were: abdominal pain/tenderness, persistent vomiting (≥2 consecutive days), clinical fluid accumulation (pleural effusion or ascites on examination or radiography), mucosal bleed, liver enlargement, and increase in hematocrit concurrent with rapid decrease in platelet count (interpreted as any hematocrit ≥20% over baseline with platelet <50000/mm^3^). Lethargy was not used as it was not routinely recorded in the dengue care path. For severe dengue, the criteria were: for severe plasma leakage, either clinical fluid accumulation or evidence of plasma leakage (hematocrit change of ≥20%) with at least one of tachycardia (pulse >100/minute), hypotension (systolic blood pressure <90 mmHg), or narrow pulse pressure (<20 mmHg); severe bleeding was defined as WHO Grade 2 or above: hematemesis, melena, menorrhagia or clinical drop in hemoglobin requiring whole blood or packed red cell transfusion; severe organ involvement comprised hepatic injury (aspartate [AST] or alanine transaminase [ALT] levels ≥1000 unit/L), renal impairment (Stage 2 Acute Kidney Injury [19] defined as serum creatinine increase of 100% over baseline or calculated norm for age/gender/race), or impaired consciousness. No dengue-related myocarditis was found in this cohort.

### Statistical analysis

For descriptive analysis, Chi-square and Fisher's exact tests were used for categorical variables, and t-test and Mann–Whitney U tests for continuous variables. Inter-rater agreement was compared using Cohen's kappa and marginal homogeneity using Bhapkar's coefficient of concordance. Sensitivities of guidelines were compared using McNemar's test for the difference between correlated proportions. Length of hospitalization was reported using geometric means and interquartile ranges calculated as for Tukey's hinges. Differences between severity categories were compared using Kruskall-Wallis test. Statistical analyses were performed using IBM SPSS Statistics 19 (SPSS Inc, 2010).

## Results

### Demographic and clinical features

Our cohort had a median age of 33 years (range 13–84 years). There was a male predominance overall as previously reported for Singapore [20]. Race distribution was similar to the national ethnic profile in both years. Hematological and biochemical parameters were statistically different betwen 2007 and 2004, as shown in Table 1. This may be of limited clinical significance as the absolute differences were small.

**Table 1:** Demographic, laboratory, treatment and outcome data of laboratory confirmed adult dengue cases.

		2004 cohort	2007cohort	Total	p-value
Number of cases		917	361	1278	
Age, median (range), years		32 (13–77)	35 (14–84)	33 (13–84)	<0.001
Male		619 (67.5%)	258 (71.5%)	877 (68.6%)	0.181
Race					0.008
	Chinese	698 (76.1%)	250 (69.3%)	948 (74.2%)	
	Indian	79 (8.6%)	39 (10.8%)	118 (9.2%)	
	Malay	52 (5.7%)	16 (4.4%)	68 (5.3%)	
	Others	88 (9.6%)	56 (15.5%)	144 (11.3%)	
Singaporean		564 (61.5%)	152 (42.1%)	716 (56.0%)	<0.001
Recent travel		16 (1.7%)	55 (15.2%)	71 (5.6%)	<0.001
Hospitalized		917 (100.0%)	318 (88.1%)	1235 (96.6%)	<0.001
Leukocyte nadir, median (range), ×10^9^/L		2.1 (0.6–8.2)	2 (0.8–7.2)	2.1 (0.6–8.2)	0.544
Platelet nadir, median (range), ×10^9^/L		41 (2–206)	34 (4–267)	39 (2–267)	<0.001
Maximum hematocrit, median (range), %		44.8 (28.3–55.4)	45.4 (30.6–58.2)	45 (28.3–58.2)	0.005
Maximum serum creatinine, median (range), mmol/L		77 (33–303)	87 (44–182)	80 (33–303)	<0.001
Maximum alanine transaminase, median (range), unit/L		71 (9–4018)	60.5 (11–2315)	67 (9–4018)	<0.001
Maximum aspartate transaminase, median (range), unit/L		112 (13–12541)	98.5 (16–4850)	107.5 (16–12541)	0.012
Intravenous fluid administration		635 (69.2%)	314 (87.0%)	944 (74.3%)	<0.001
Platelet transfusion		124 (13.5%)	33 (9.1%)	157 (12.3%)	0.036
Packed red cell transfusion		2 (0.2%)	3 (0.8%)	5 (0.4%)	0.140
Length of stay (LOS), mean (interquartile range), days		4.96 (4–6)	4.82 (4–6)	4.92 (4–6)	0.434
Intensive care unit (ICU) admission		4 (0.4%)	2 (0.6%)	6 (0.5%)	0.677
ICU LOS, median (range), days		2.5 (1–5)	2.5 (2–3)	2.5 (1–5)	>0.999
Death		1 (0.10%)	0 (0%)	1 (0.10%)	>0.999

Among the 1278 patients, intravenous fluid was administered to 944 (74.3%) and platelet transfusion in 157 (12.3%). The median length of hospital stay was 5 days (range 2–22 days).

### Length of hospitalization, intensive care admission and death

Using independent clinical gold standards of severity, we examined the performance of WHO 1997 and 2009 compared with length of hospital stay, the requirement for intensive care and death. Six cases required intensive care including the single fatality in this series. Two of these cases did not qualify as DHF by WHO 1997 criteria: both had no bleeding manifestations (although tourniquet tests were not performed); one developed fulminant hepatic failure with encephalopathy and the other had dengue encephalopathy. Of the four DHF cases, two had DSS. Severe dengue criteria captured all six cases, of which four had severe plasma leakage, two severe bleeding and three severe organ impairment. The single mortality had only a history of peptic ulcer disease, and experienced a rapid progression to death 24 hours after hospitalization. This case fulfilled DSS criteria and had severe plasma leakage and organ impairment (liver and kidney) by WHO 2009 criteria but did not have severe bleeding.

As the number of patients with ICU admission and death was low in this study, we determined the clinical significance of WHO 1997 and 2009 classifications by examining hospital length of stay (Table 2). Inpatients were admitted for a mean of 4.92 days, with no significant difference between the 2004 and 2007 cohorts (p = 0.135). No significant difference was seen in the length of hospitalization among cases with DF, DHF and DSS although there was a trend of increasing length of hospital stay with increasing severity. With WHO 2009 classification, severe dengue cases stayed longer (p<0.001) which was borne out in both years. This was mainly due to cases with severe plasma leakage staying longer than those with severe bleeding or organ impairment (p<0.001).

**Table 2:** Length of hospitalization by year and World Health Organization (WHO) dengue severity classification.

		Mean length of hospitalization (interquartile range), days			
		2004 cohort	2007 cohort	Total	p-value^a^
	All cases	4.96 (4–6)	4.82 (4–6)	4.92 (4–6)	
WHO 1997 classification	Dengue Fever (non-DHF confirmed dengue)	4.96 (4–6)	4.56 (4–6)	4.90 (4–6)	0.224
	Dengue Hemorrhagic Fever	5.10 (4–6)	4.99 (4–6)	5.02 (4–6)	
	Dengue Shock Syndrome	3.53 (2–5)	5.69 (5–7)	5.23 (4–7)	
WHO 2009 classification	Non-severe dengue without warning signs	4.81 (4–6)	4.59 (4–6)	4.79 (4–6)	<0.001
	Non-severe dengue with warning signs	5.04 (4–6)	4.67 (4–6)	4.88 (4–6)	
	Severe dengue	5.62 (4–7)	5.23 (4–6)	5.43 (4–6.5)	
Severe manifestations^b^	Severe plasma leakage	6.16 (5–7)	5.53 (5–6)	5.89 (5–7)	<0.001
	Severe bleeding	4.51 (3.5–5)	4.54 (4–6)	4.54 (4–5)	
	Severe organ impairment	5.28 (4–6)	4.24 (3–6)	5.21 (4–6)	

a Difference between severity categories assessed with Kruskall-Wallis test for total cohort.

b Only isolated severe manifestations were compared against each other, and cases with more than one severe manifestation were excluded from analysis.

### Comparison of WHO 1997 and 2009 criteria

In our laboratory confirmed dengue cohort, 92.5% of cases experienced symptoms, signs and laboratory abnormality fulfilling WHO 1997 dengue fever (DF) criteria, and 99.3% by WHO 2009 probable dengue criteria. The 6.8% increase in sensitivity of the WHO 2009 clinical case definition over the equivalent WHO 1997 criteria was statistically significant (p<0.001).

We classified our cohort using WHO 1997 and 2009 classifications in Table 3. The two sets of criteria are in poor agreement, as measured by the low Cohen's kappa of 0.1999 and the significant difference in marginal homogeneity (Bhapkar test p<0.001). The five DSS cases classified as non-severe dengue without warning signs had hypoproteinemia as the sole indication of plasma leakage, which qualified only with WHO 1997 criteria. Importantly, as evidence of shock, they all had hypotension although none had narrow pulse pressure; only one had tachycardia. All demonstrated systolic blood pressures of ≥100–120 mmHg after recovery.

**Table 3:** World Health Organization 1997 versus 2009 three-category classification of dengue based on 1278 adult cases confirmed by polymerase chain reaction.

	Non-severe dengue without warning signs (%)	Non-severe dengue with warning signs (%)	Severe dengue (%)	Total (%)
Dengue fever (non-DHF confirmed dengue)	608 (57.3)	346 (32.5)	107 (10.8)	1061 (83)
Dengue haemorrhagic fever (DHF Grades 1 and 2)	11 (6)	102 (55.7)	70 (38.3)	183 (14.3)
Dengue shock syndrome (DHF grades 3 and 4)	5 (14.7)	10 (29.4)	19 (55.9)	34 (2.7)
Total	617 (48.3)	457 (35.8)	204 (16.0)	1278 (100)

The other discordant cell consisted of cases classified as non-DHF by WHO 1997 criteria, but as severe dengue by WHO 2009 criteria. Of 107 cases, 16 (14.9%) had isolated elevated aminotransferases ≥1000 unit/L without plasma leakage or hemorrhagic manifestations. All had rapid improvement during hospitalization and none had hepatic encephalopathy. Twenty (18.7%) had isolated gastro-intestinal bleeding with no evidence of plasma leakage or shock; of these none had a drop in haemoglobin below 8 g/dL and none required blood transfusion. Sixty (56.1%) had evidence of plasma leakage with isolated tachycardia and no hemorrhagic manifestations. The remainder (11 cases) had more than one category of severe dengue.

Of 204 severe dengue cases, 11.3% had more than one severe manifestation. Sixty two percent had severe plasma leakage, 31% had severe bleeding and 18% had severe organ involvement. Of those with isolated severe plasma leakage, 55% had tachycardia as the only criterion for shock. Of those with isolated severe bleeding, 79% had no corroborative clinical features of severity (hypotension, haemoglobin <8 g/dL, blood transfusion given). Of those with isolated severe elevated aminotransferases (n = 20), none fulfilled criteria for acute liver failure (encephalopathy and coagulopathy).

WHO 2009 recommends that all cases of severe dengue and dengue with warning signs be hospitalised; this would lead to at least 51.3% hospitalization of our cohort. In comparison, hospitalization of DHF and DSS cases based on WHO 1997 would lead to a 17.0% hospitalization rate.

### Differences between epidemics

We found a marked difference in the severity of cases between the two epidemics using either WHO 1997 or 2009 criteria. In the 2004 epidemic, 11% of cases were severe dengue and 6% were DHF. In the 2007 epidemic, 27% of cases were severe dengue and 43% were DHF. The distribution of severe manifestations was different, with significantly more organ impairment (mainly elevated aminotransferases) in 2004, and significantly more severe bleeding in 2007 (Table 4).

**Table 4:** Manifestations of severe dengue in 2004 vs 2007 cohorts.

	Number of cases (% of total severe dengue)		
	2004 cohort	2007 cohort	p-value
Severe dengue	105 (100%)	99 (100%)	-
Severe plasma leakage	65 (62%)	64 (64%)	0.771
Severe bleeding	14 (13%)	49 (49%)	<0.001
Severe organ impairment (total)	31 (30%)	6 (6%)	<0.001
Liver impairment	29 (28%)	4 (4%)	<0.001
Renal impairment	2 (2%)	2 (2%)	>0.999
Encephalopathy	1 (1%)	1 (1%)	>0.999

## Discussion

We found that the WHO 2009 probable dengue criteria based solely on clinical and simple laboratory criteria missed 0.7% in our laboratory-confirmed cohort. In comparison the WHO 1997 criteria missed 7.5%. This was likely due to the addition of nausea and vomiting, and the presence of any warning signs which include abdominal pain. These high sensitivities above 90% are in line with previous descriptions of Singaporean adults presenting with acute febrile illness [21], [22]. As this study was not planned to describe fully the test characteristics of both sets of clinical criteria, it will be important to assess the specificity of the new broader clinical case definition in a prospective study to aid triage of cases in resource-limited dengue endemic countries where diagnostic tests are not easily available.

The DHF/DSS criteria have been criticized for not covering the full spectrum of dengue disease and failing to categorize all cases with severe outcomes [23], [24]. In cases requiring intensive care or resulting in death, DHF criteria missed two cases in our cohort where no bleeding manifestations were present (though a formal tourniquet test was not performed) but resulted in fulminant liver failure or encephalopathy, which would be categorised as an unusual dengue manifestation not fulfilling DHF/DSS in the WHO 1997 classification. WHO 2009 thus provides clinical utility in proposing definitions for non-DHF manifestations of severe dengue. Both these cases had evidence of plasma leakage which would have fulfilled the definition in the WHO SEARO 2011 guidelines. In a separate analysis we found no cases of liver failure despite prevalent elevation of liver aminotransferases, and a lack of a threshold value of AST or ALT that correlated with severe dengue [25].

Hypoproteinemia suffices as evidence of plasma leakage in WHO 1997 but not WHO 2009 criteria. In our cohort, 49% had hyproproteinemia, with 79.3% of these not demonstrating a hematocrit change of ≥20%. It is likely that these DHF cases were on the milder end of its clinical spectrum and timely intravenous fluid therapy may have prevented a hematocrit rise of ≥20%. However, the level at which hypoproteinemia reflects the pathophysiology of plasma leakage leading to severe disease is not well-defined and merits further evaluation. A comprehensive evaluation of surrogates for vascular permeability using as a gold standard sensitive imaging modalities such as ultrasound and magnetic resonance imaging will be required to evaluate the utility of hypoprotenemia and other markers of plasma leakage, which the authors are planning.

Triage is a critical and resource-limited stage in dengue management. The use of WHO 2009 criteria for hospitalization of all cases with warning signs would like to a higher rate of hospitalization than that considering only hospitalization of DHF and DSS cases amounting to an absolute increase in hospitalization proportion of 34.3% (95% CI 31.6–37.0%).

The increase in disease severity in 2007 may be related to changes in triage of dengue cases for admission. During large outbreaks in 2004 and 2005, 80% of adult dengue cases were hospitalized [26] putting a high burden on the limited bed capacity of healthcare facilities. Subsequently, evidence-based admission criteria were developed leading to more cases being treated in primary care or in the outpatient setting [27]–[29]. We estimated that our prognostic algorithms could reduce the caseload of hospitalized mild dengue by 43.9%–56.7% [30], [31] and found an actual decrease in hospitalisation rate of 91.9% in 2006 to 53.5% in 2008 [32]. This may be reflected in the more severe hematological and biochemical parameters shown in Table 1.

Limitations of our study included its retrospective design which may have incomplete recording of signs such as petechiae and the lack of routine administration of the tourniquet test in local practice, although a standardized clinical care path and a rigorous data management protocol mitigated inaccuracy in data collection. In addition there were few truly severe cases needing ICU care and only one death, which limited our ability to assess the clinical relevance of both dengue classifications. Our patient cohorts were adult patients who presented to hospital and were admitted for inpatient care in a country hyperendemic for dengue, limiting generalizability to pediatric populations, presentations at primary care and countries with a lesser degree of dengue endemicity. Drawing more detailed conclusions regarding dynamic impact on clinical management, decision making and resource utilization are urgent questions which will be better assessed using a prospective cohort and advanced modelling techniques which are beyond the scope of this paper.

Despite these limitations, our study comprised a large number of dengue PCR-positive adult patients with DHF in 14.3% and severe dengue in 16.0% of the cohort. Our data highlighted the clinical utility of WHO 2009 in describing non-DHF manifestations of dengue that may be significant, and some areas that can be refined in WHO 2009 dengue classification. As plasma leakage underlies the pathophysiology of DHF and DSS, it may be prudent to retain hypoproteinemia in the definition of plasma leakage in addition to hematocrit change and clinical fluid accumulation, and combine narrowing of pulse pressure and tachycardia in the definition of shock in addition to systolic hypotension. Clinically severe bleeding could be defined as bleeding associated with systolic hypotension, hemoglobin <8 g/dL or a drop of hemoglobin more than 2 g/dL, or requiring blood transfusion. Finally, in the absence of dengue-specific definitions of organ failure, standard definitions of liver and renal impairment may be adopted, namely using the definition of acute liver failure recommended by the American Association for the Study of Liver Diseases [33], and using the definitions of acute kidney injury from the Acute Kidney Injury Network [19]. Lack of such quantification in the WHO 2009 definitions hinders global standardization of definitions; we have transparently stated our parameters based on prior publications as stated and local norms.

In conclusion, the WHO 2009 classification shows discordance with the WHO 1997 classification for clinical diagnosis and disease severity. The WHO 2009 guideline highlighted aspects of dengue disease previously outside of the DF/DHF/DSS classification, previously classified as unusual manifestations. Definitional issues with regard to severity of dengue disease remain, particularly in the adult population. Our report on the utility and pitfalls of the new scheme will be constrained by our study population which is an adult cohort referred to a university teaching hospital at an early stage of illness (while still PCR positive) in a dengue hyperendemic country during two predominant DENV-1 and DENV-2 outbreaks. It should be extended to different populations given the wide spectrum of dengue disease.
